# Application of a modified clinical classification for pulmonary arterial hypertension associated with congenital heart disease in children: emphasis on atrial septal defects and transposition of the great arteries. An analysis from the TOPP registry

**DOI:** 10.3389/fcvm.2024.1344014

**Published:** 2024-02-02

**Authors:** Julie Wacker, Tilman Humpl, Rolf M. F. Berger, Dunbar Ivy, David Bowers, Damien Bonnet, Maurice Beghetti

**Affiliations:** ^1^ Pediatric Cardiology Unit, Department of Women, Child and Adolescent, Children’s University Hospital, and University of Geneva, Geneva, Switzerland; ^2^ Pediatric Cardiology and Critical Care Medicine, The Hospital for Sick Children University of Toronto, Toronto, ON, Canada; ^3^ Centre for Congenital Heart Diseases, Pediatric Cardiology, Beatrix Children’s Hospital, University Medical Center Groningen, University of Groningen, Groningen, Netherlands; ^4^ Pediatric Cardiology, Children’s Hospital Colorado, University of Colorado School of Medicine, Aurora, CO, United States; ^5^ School of Allied Health Sciences, University of Suffolk, Ipswich, United Kingdom; ^6^ Centre de Référence Malformations Cardiaques Congénitales Complexes, M3C-Necker Hospital for Sick Children, Assistance Publique des Hôpitaux de Paris, University of Paris Cité, Paris, France

**Keywords:** pulmonary arterial hypertension, pulmonary hypertension, congenital heart disease, Eisenmenger syndrome, pediatrics, atrial septal defect, transposition of the great arteries, classification

## Abstract

**Aims:**

A proportion of patients with pulmonary arterial hypertension associated with congenital heart disease (PAH-CHD) do not fit in the current classification. We aimed to analyse the applicability of an adapted clinical classification of PAH-CHD to pediatric patients using the TOPP-1 registry (Tracking Outcomes and Practice in Pediatric Pulmonary Hypertension) and focus on atrial septal defects (ASD) and transposition of the great arteries (TGA).

**Methods and results:**

Hemodynamic and clinical data of all patients with PAH-CHD in the TOPP cohort were reviewed. Patients were classified according to predefined ABCDE categories (A: Eisenmenger syndrome, B: left-to-right shunt, C: coincidental defects, including all ASDs, D: corrected CHD, E: TGA), or as complex CHD (group 5), by 2 independent investigators. In case of disagreement, a third reviewer could either settle a final decision, or the patient was deemed not classifiable. Survival curves were calculated for each group and compared to idiopathic PAH patients of the registry. A total of 223 out of 531 patients in the registry had PAH-CHD, and 193 were categorized to the following groups: A 39(20%), B 27(14%), C 62(32%) including 43 ASDs, D 58(30%), E 7(4%), whereas 6 patients were categorized as group 5, and 10 patients were unable to be classified. No survival difference could be demonstrated between the groups.

**Conclusions:**

This modified classification seems to be more applicable to pediatric PAH-CHD patients than the previous classification, but some patients with PAH-CHD who never had a shunt remain unclassifiable. The role of ASD in pediatric PH should be reconsidered.

## Introduction

Pulmonary hypertension (PH) is a serious complication in some types of congenital heart disease, impacting patients’ morbidity and mortality. Pulmonary arterial hypertension associated with congenital heart disease (PAH-CHD) and idiopathic pulmonary arterial hypertension (iPAH) are the two main causes of pulmonary arterial hypertension (PAH) in children.

PH classification is evolving with improved knowledge in the pathobiology, epidemiology and phenotyping of the disease. The current PH classification intends to categorize multiple conditions into five groups according to similarities in clinical presentations, pathological findings, hemodynamic characteristics, and treatment strategies (1, 2). PAH-CHD belongs to group 1 of the classification, alongside other causes of PAH, such as iPAH.

A clinical classification of congenital systemic-to-pulmonary shunts first appeared in the European guidelines in 2003 (3), and was refined in 2009, identifying four main phenotypes of congenital systemic-to-pulmonary shunts associated with PAH (A: Eisenmenger's syndrome, B: PAH associated with systemic-to-pulmonary shunts, C: PAH with small defects and D: PAH after corrective cardiac surgery) (4). This PAH-CHD classification, developed for adult patients and considered adaptable to children, was further updated during the 5th World Symposium on PH in Nice in 2013 (5, 6). However, this classification only considers patients with systemic-to-pulmonary shunts. Pediatric patients with PAH-CHD are a very heterogeneous group, including many children with complex heart defects or those having PH and CHD in the absence of a shunt (7). Indeed, a substantial proportion of pediatric patients proved to be not-classifiable according to the Nice CHD classification (8). The addition of a subgroup of complex congenital heart disease in group 5 of the PH classification (« PH with unclear and/or multifactorial mechanisms ») at the 6th World Symposium on Pulmonary Hypertension (WSPH) in Nice, France, in 2018 allowed the classification of segmental disorders, single ventricle physiology and scimitar syndrome, although “complex CHD” group does not appear anymore in the most recent set of European guidelines (2).

Despite those changes, the current predefined categories still do not allow proper classification for all pediatric patients with PAH and CHD and these “unclassifiable” patients deserve some specific consideration.

Firstly, pediatric patients with an atrial septal defect (ASD) and PAH form an intriguing group. As per current recommendations, patients with a large (>2 cm) ASD should be classified in the group B of PAH-CHD (PAH associated with prevalent systemic-to-pulmonary shunts). However, natural history studies of ASD show that PAH normally does not occur before the 3rd or 4th decade (9, 10). In ASD, the most frequent pre-tricuspid shunt, pulmonary blood flow will increase, but without transmission of the systemic pressure into the PA, which will cause much less damage on the lung vasculature than a post-tricuspid shunt, like a ventricular septal defect, because the lung's circulation is not efficient at normalizing shear stress with increased flow and high pressure (11).

Secondly, patients with PAH after neonatal arterial switch operation for transposition of the great arteries (TGA), in the absence of haemodynamically relevant residual lesions, is an increasingly recognised entity, occurring in about 1% of timely operated patients (12). Due to neonatal repair in most patients, these patients are difficult to classify in the current shunt-based PAH-CHD classification as they do not have a long-standing shunt lesion.

A valid classification is able to identify patient groups with similar disease characteristics, risks, or outcomes, allowing a tailored treatment approach to the individual patient. Whereas differences in disease severity and survival between PAH-CHD groups defined as per the Nice CHD classification have been demonstrated in adults (13), the prognostic value of this classification in children remains to be proven. A recent analysis from the Tracking Outcomes and Practice in Pediatric Pulmonary Hypertension (TOPP) registry showed that patients with an open shunt (whether unrepaired or residual) had a better survival than all the other categories of PAH (14). However, no analysis of the different PAH-CHD subgroups was performed. We sought to apply a modified version of the clinical PAH-CHD classification that relocates patients with any size ASD, and patients with TGA to the TOPP registry and assess if this improves the classification of pediatric PAH-CHD and predicts outcome.

## Methods

A modified classification of PAH-CHD for pediatric patients was established for the purpose of the study and is summarized in Table 1. The two main modifications compared to the current classification were: (1) all ASDs were classified in the coincidental shunt group C; and (2) addition of a group E for repaired TGA. This was decided after discussion and consensus among experts in the field.

**Table 1:** Comparison of the classical PAH-CHD classification with the ABCDE classification.

	Guidelines Galiè 2015		ABCDE classification
Eisenmenger's syndrome	Includes all large intra- and extra-cardiac defects which begin as systemic-to-pulmonary shunts and progress with time to severe elevation of PVR and to reversal (pulmonary-to-systemic) or bidirectional shunting; cyanosis, secondary erythrocytosis, and multiple organ involvement are usually present	A	Patients with previous left to right shunt who present with a right to left shunt and saturation ≤ 92% at the time of diagnosis. This includes patients below 0.5-1 year with Eisenmenger physiology who never had a left-to-right shunt
PAH associated with prevalent systemic-to-pulmonary shunts	Correctable Non correctable Includes moderate to large defects; PVR is mildly to moderately increased, systemic-to-pulmonary shunting is still prevalent, whereas cyanosis at rest is not a feature	B	Patients with left-to-right shunt but saturation > 92% (independent of operability)
PAH with small/coincidental defects	Marked elevation in PVR in the presence of small cardiac defects (usually VSD < 1 cm and ASD < 2 cm of effective diameter assessed by echo), which themselves do not account for the development of elevated PVR; the clinical picture is very similar to idiopathic PAH. Closing the defects is contra-indicated	C	Patients with so called « coincidental shunts », for example small VSD and/or PDA. This includes all pediatric patients with ASD.
PAH after defect correction	Congenital heart disease is repaired, but PAH either persists immediately after correction or recurs/develops months or years after correction in the absence of significant postoperative haemodynamic lesions.	D	Patients with operated CHD without haemodynamically significant residual shunts
		E	Patients with TGA, who underwent neonatal ASO
Classification outside ABCDE			
Group 5^a^	Patients with segmental PH incl. TOF/MAPCAs Patients with Scimitar syndrome (complex CHD) Patients with single ventricles		** **
Group 1	iPAH		** **

ASD, atrial septal defect; CHD, congenital heart defect; iPAH, idiopathic pulmonary arterial hypertension; PAH, pulmonary arterial hypertension; PDA, patent ductus arteriosus; PVR, pulmonary vascular resistance; VSD, ventricular septal defect.

^a^ Patients with complex CHD have not been included in the latest guidelines.

The TOPP-1 registry is a centre-based registry initiated January 31, 2008, covering 31 centres in 19 countries. The TOPP registry collects data on the assessment, treatment, and follow-up of pediatric PH patients (15). Participating centres include consecutive patients between 3 months and 18 years of age presenting with PAH or with PH groups 3–5 (classified according to the 2003 Third World Pulmonary Hypertension Symposium) and diagnosed on or after January 1, 2001. Patients are eligible for TOPP inclusion when meeting pre-specified hemodynamic criteria (mean pulmonary arterial pressure (mPAP) ≥ 25 mm Hg, pulmonary vascular resistance index (PVRi) ≥ 3WU*m^2^, and mean pulmonary capillary wedge pressure ≤12 2 mmHg). The cut-off of 12 mmHg was set below the 15 mmHg of the adult definition when the registry was initiated, in order to obtain a pediatric population of true PAH. Hemodynamic data of all included patients are reviewed by the TOPP registry executive board members to confirm diagnosis, leading to a cohort of heart catheterization confirmed PAH patients (PH-confirmed). Treatment strategies and follow-up are determined by individual centres, and no recommendations are made from the registry board members.

For the current study, case report forms (CRF) from every patient entered into the TOPP registry before July 2015 (with a diagnosis of PAH-CHD and iPAH) were revised independently by two different researchers (MB and TH). Subjects were classified according to the modified ABCDE classification (Table 1) or where applicable, to group 5 of the updated PH classification (complex congenital heart disease) (16, 17), based on the patient's status at diagnosis. In case of agreement between the two reviewers, the patients were classified into the designated category. In case of disagreement, a third reviewer (DB) would settle a final decision. If the third reviewer did not agree with neither of the two first reviewers, the patient was deemed unclassifiable. Patients with insufficient data provided in the registry were also identified.

The baseline variables age, sex, functional class (NYHA), comorbidities, hemodynamics and PH-targeted treatment were compared across the ABCDE categories. As a comparator group, patients with idiopathic PAH in the TOPP registry were included in the analysis. *P*-values were reported in the exploratory sense, using the Kruskal–Wallis test for continuous variables and the chi-squared test for categorical variables.

Survival was analysed from diagnosis to the composite endpoint of transplantation or death. Patients with no discontinuation event and no follow-up visits were excluded from the survival analysis. The ABCDE and iPAH groups were compared using Kaplan–Meier survival curves, with right censoring applied if patients discontinued for other reasons. The log rank test was used to assess statistical significance of the difference in survival between the groups.

Statistical analyses were conducted using IBM SPSS Statistics version 27. No imputations were used for missing data, and all analyses were carried out using available data only.

## Results

### Patients' characteristics

At the data cut off (July 2015), the TOPP-1 registry included 531 pediatric patients with confirmed PH.

Of these, 223 were diagnosed PAH-CHD and fulfilled inclusion criteria and 186 (83%) of these could be classified according to the previous ABCD classification. Seven patients with TGA after neonatal arterial switch surgery were classified as new group E, 6 patients were classified in group 5, whereas 10 patients (4.5%) remained unclassifiable with the modified ABCDE classification. They had adequately corrected left-sided lesions, (pulmonary vein stenosis, mitral stenosis and coarctation of the aorta) with a pulmonary capillary wedge pressure below 12 mmHg, fulfilling the diagnosis of PAH.

The 14 remaining patients could not be classified because of insufficient data in the CRF.

**Figure 1:**
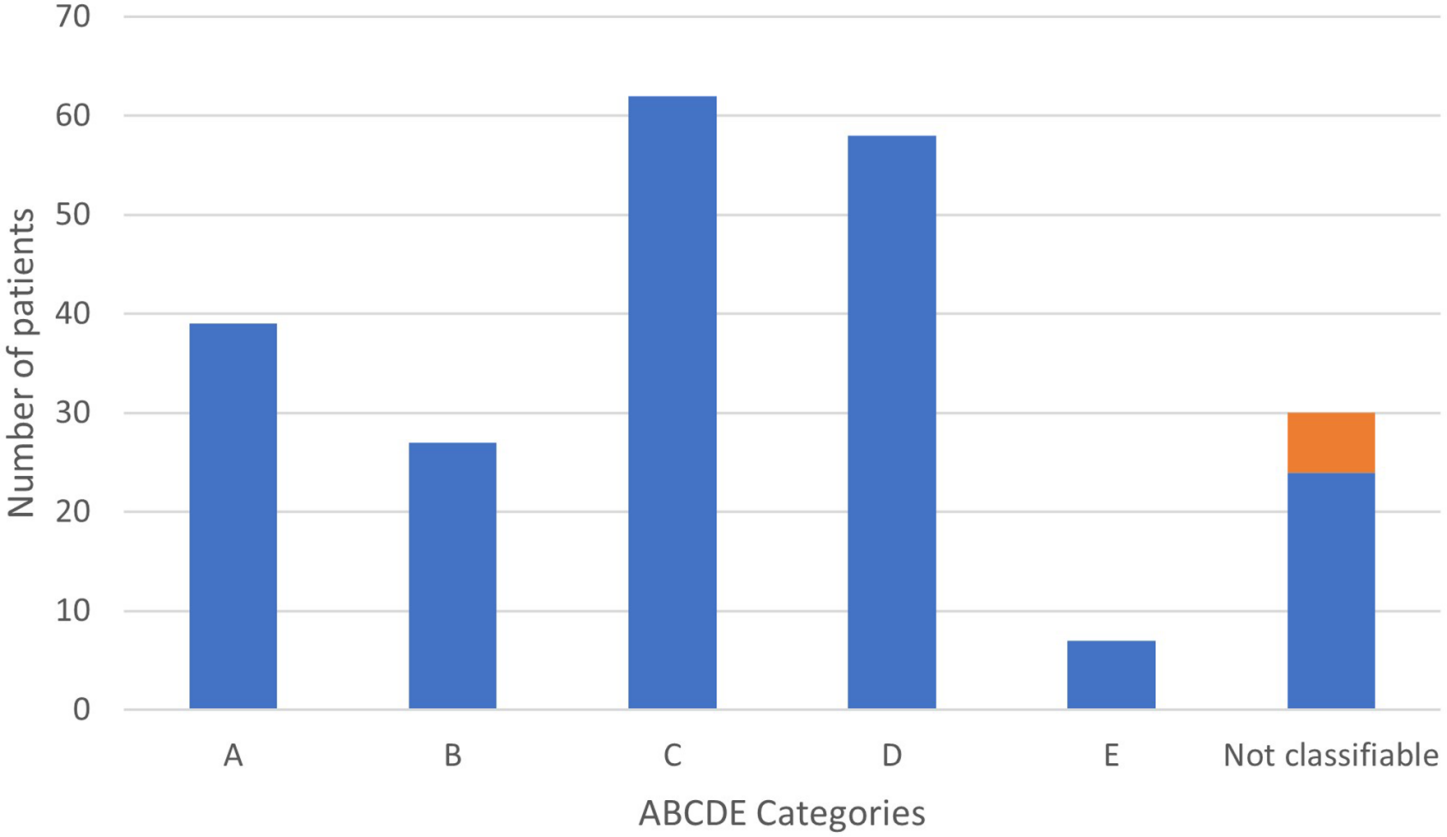
Distribution of children with PAH-CHD according to ABCDE classification. In orange, previous « group 5 ».

Figure 1 shows the distributions of PAH-CHD patients across the various groups. Of the 193 classifiable patients in the modified ABCDE categories, children with coincidental shunts (62 patients, 32%) accounted for the most prevalent group followed by postoperative PAH (58 patients, 30%) and Eisenmenger syndrome (39 patients, 20%). A minority of children had PAH with left to right shunt (27 patients, 14%), and TGA after neonatal arterial switch surgery (7 patients, 4%). Of note, 43/62 patients in group C had an ASD, the others had very small VSDs.

Table 2 summarizes demographic, functional, and hemodynamic characteristics of patients across the ABCDE subgroups. Median age at diagnosis was higher in groups A and D, and lower in group E. The majority of patients were in functional class II and III, independently of the CHD group. Eisenmenger patients had the highest mPAP and PVRi. Group C had similar hemodynamics compared to iPAH patients.

**Table 2:** Demographical and clinical characteristics of patients across ABCDE categories and iPAH.

Group	A	B	C	D	E	iPAH	*p*-value (exploratory)
Number of patients	39	27	62	58	7	237	
Age at diagnosis [years]	10 (2-14)	5 (2–10)	4 (1–11)	7 (4–13)	0 (0–1)	7 (4–12)	<0.001 (KW)
Female [*n*, %]	28 (72%)	17 (63%)	29 (47%)	31 (53%)	1 (14%)	146 (62%)	0.017 (Chi)
Comorbidities:							
Trisomy 21	13 (33%)	6 (22%)	11 (18%)	14 (24%)	0	3 (1%)	<0.001 (Chi)
Prematurity	4 (10%)	3 (11%)	9 (15%)	7 (12%)	0	20 (8%)	0.678 (Chi)
Residence >1,500 m	1 (3%)	1 (4%)	7 (11%)	8 (14%)	0	n/a	
Syncope [*n*, %]	4 (10%)	1 (4%)	6 (10%)	13 (22%)	0	83 (35%)	<0.001 (Chi)
NYHA FC							<0.004 (Chi)
I	2 (5%)	3 (11%)	10 (16%)	11 (19%)	2 (29%)	32 (14%)	
II	16 (41%)	14 (52%)	33 (53%)	33 (57%)	4 (57%)	90 (38%)	
III	18 (46%)	10 (37%)	19 (31%)	13 (22%)	1 (14%)	83 (35%)	
IV	3 (8%)	0	0	1 (2%)	0	31 (13%)	
O2 saturation [%]	84.6 ± 10.2	95.2 ± 3.0	92.3 ± 7.5	96.9 ± 2.6	98.0 ± 2.3	96.2 ± 4.2	<0.001 (KW)
mPAP [mmHg]	67.5 ± 15.8	60.1 ± 18.9	57.0 ± 17.1	53.1 ± 21.5	49.9 ± 15.5	59.3 ± 18.0	<0.001 (KW)
PVRi [WU*m^2^]	20.3 ± 12.8	10.7 ± 6.4	17.0 ± 13.5	14.7 ± 11.0	13.0 ± 7.9	17.8 ± 10.7	<0.001 (KW)
PVRi/SVRi	1.2 ± 0.7	0.6 ± 0.6	1.2 ± 1.6	0.7 ± 0.3	0.8 ± 0.4	0.9 ± 0.3	<0.001 (KW)
PCWP [mmHg]	8.0 ± 2.6	9.4 ± 2.5	7.7 ± 2.9	9.4 ± 2.5	8.9 ± 2.0	8.4 ± 2.6	0.007 (KW)
Cardiac Index [L/min/m^2^]	4.0 ± 2.0	3.6 ± 1.3	3.8 ± 2.8	3.6 ± 1.8	3.9 ± 3.6	3.4 ± 1.4	0.404 (KW)
RA pressure [mmHg]	6.0 ± 4.1	6.5 ± 2.7	6.7 ± 3.6	8.6 ± 3.5	6.7 ± 3.1	7.0 ± 3.9	0.002 (KW)

Data expressed as median (IQR) or mean ± SD.

Chi, chi-squared test; KW, Kruskal–Wallis test; mPAP, mean pulmonary artery pressure; PCWP, pulmonary capillary wedge pressure; PVRi, pulmonary vascular resistance indexed; RA, right atrial.

At inclusion, 148 patients across the ABCDE subgroups were treated with pulmonary vasodilators (77%), and 56 (29%) were treated with combination therapy including 12 (6%) on triple therapy. 16 patients (8%) were on parenteral prostanoids.

In the comparison group, data from 237 iPAH patients included in the TOPP registry were analysed. The median age at the time of inclusion was 7 (IQR 4–12) years, with 146 (62%) females. In this population, 205 (86%) patients were treated with approved PAH-specific medications, with 112 (47%) with combination therapy, including 44 (19%) on triple therapy. 64 patients (27%) were on parenteral prostanoids.

### Survival analysis

There were 18 transplantations and 72 deaths during a mean (SD) follow up of 61 (42) months, with 12 transplantation and 44 deaths occurring in the iPAH group, and 6 transplantations and 28 deaths across the ABCDE categories. Median (IQR) follow-up time was 58 (23–88) months. In the overall PAH-CHD population, 1-, 3- and 5- year death and transplantation-free survival was 93.1%, 86.5% and 82.0%. According to the groups, 1 and 5 year survival were 97.3% and 94.3% for group A; 100% and 95.0% for group B; 92.6% and 83.0% for group C; 94.6% and 79.4% for group D; 100% and 100% for group E; 91.2% and 78.7% for iPAH. By comparison, Group 5 survival was 75% at 1 year and 75% at 5 years, based on a small cohort with only one early death.

**Figure 2:**
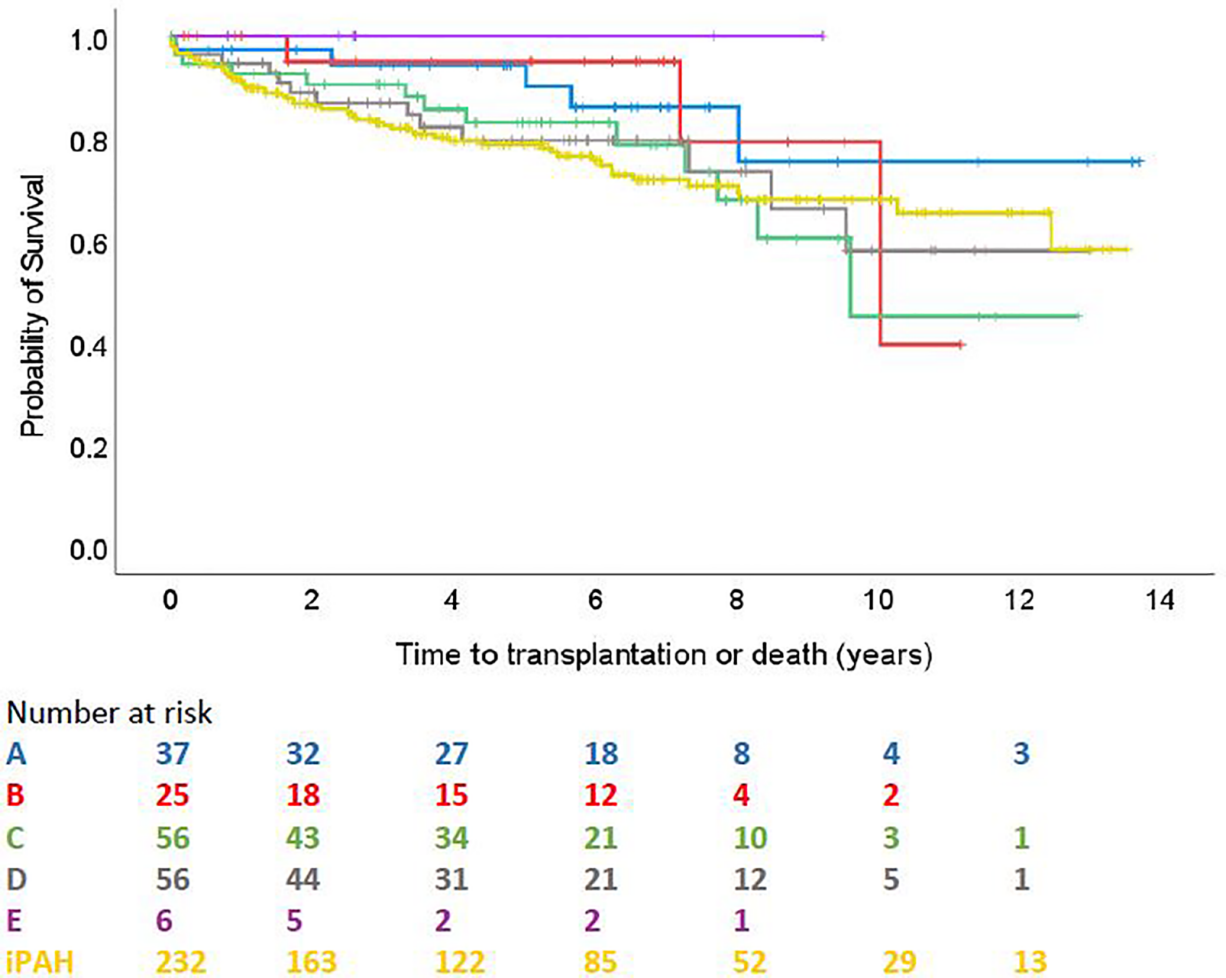
Time to transplantation or death across ABCDE categories and iPAH (PH confirmed patients with ≥1 follow-up visit).

**Figure 3:**
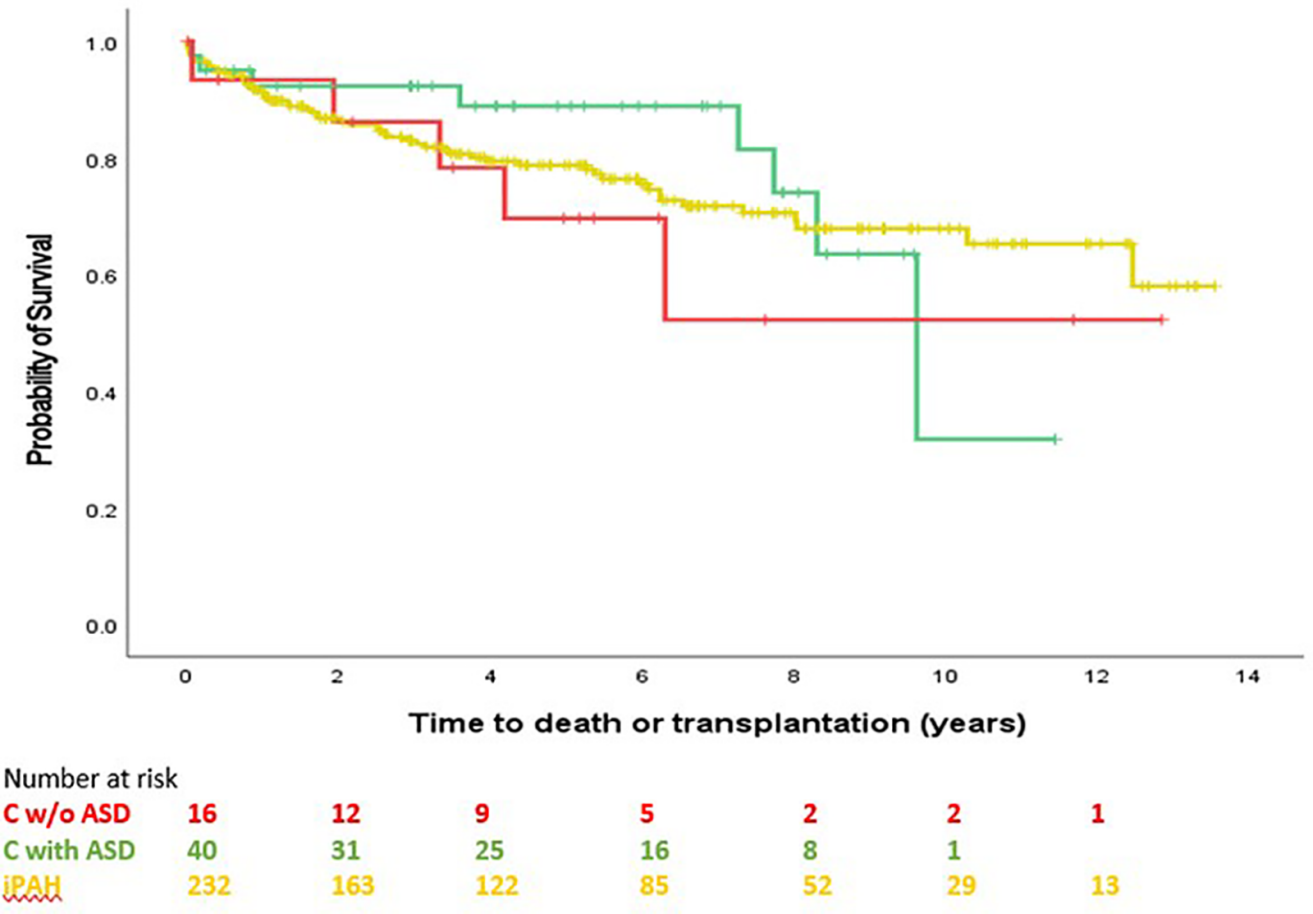
Time to transplantation or death, C without ASD, C with ASD, pure iPAH (PH confirmed patients with ≥1 follow-up visit). No significant difference between groups (*p* = 0.653).

Survival comparison between groups is illustrated in Figure 2. No survival comparison between any of the groups reached statistical significance. Figure 3 compares survival between group C with ASD, group C without ASD and iPAH. There was no statistically significant difference between those 3 groups.

## Discussion

The main findings of this study are: (1) The proposed modified clinical classification of PAH-CHD is applicable to the majority of the children from the TOPP registry, but some patients who never had a shunt, mainly adequately repaired left-sided lesions, remain unclassifiable. (2) This clinical classification was not able to show a difference in survival between the groups.

### Baseline characteristics of patients

We observed a female predominance in almost all groups, except in group C (where there was near-balance), and group E (with only one female patient). Female preponderance in PAH, also in association with CHD, has been reported in adults (18, 19) and in the whole cohort of the TOPP registry (15). The different sex distribution in group E can be explained by the male preponderance in incidence of babies born with TGA (20).

Patients were different in terms of age at diagnosis throughout ABCDE categories. Young age at diagnosis was notable in group E (patients with repaired TGA), with most patients diagnosed during the first year of life. However, the oldest patient of this group was diagnosed at 11 years of age, illustrating the possibility of PH to become clinically evident many years after corrective TGA surgery (21).

Children with trisomy 21 were overrepresented in the ABCDE groups compared to iPAH patients. Children with Down syndrome are known to have CHD in over 40% of cases and moreover a multifactorial PH etiology (PAH-CHD, developmental lung disease, upper airway obstructive disease, …) (22). Groups did not differ for other comorbidities known to influence PH, e.g, prematurity, or residence at high altitude.

Most patients were in NYHA functional class II or III, independent of ABCDE subgroup, which was similar to the iPAH comparison group and reflects the distribution in the whole registry (15).

Eisenmenger patients had the highest mPAP and PVRi, despite similar functional class distribution.

### Applicability of the adapted ABCDE classification

The PAH-CHD classification was initially proposed for adult patients with systemic to pulmonary shunts (3). Pediatric patients, although representing a minority amongst all PH patients, form a heterogeneous group, with a variety and complexity of CHD which cannot be reduced to the notion of a shunt. Moreover, the growing population of adults with CHD might benefit from a refinement of the classification taking into account more complex CHD. In a cohort of 117 children with PAH-CHD, Zijlstra et al. demonstrated that 15 (11%) of the patients did not fit any of the 2013 Nice CHD groups (8). Some unclassifiable patients of this cohort might have been placed in a category following the introduction of complex congenital heart disease in group 5 in 2018. Nevertheless, 6 of the unclassifiable patients of their cohort were patients with TGA after neonatal repair (arterial switch operation). In light of this observation, and recognizing the challenge with this increasingly diagnosed entity, we opted to add a fifth category (E) to the existing classification, for patients with this condition.

The distribution of patients across PAH-CHD categories slightly differs from what has been reported by Zijlstra et al. (8), but our decision to reclassify all isolated ASD in the coincidental CHD group probably explains the increased prevalence of group C.

In the present study, 10 patients (4.5%) could not be classified. They had CHD and PAH, never had a shunt, but had adequately corrected left-sided lesions, including pulmonary vein stenosis, mitral stenosis and coarctation of the aorta. Their pulmonary capillary wedge pressure was below 12 mmHg, fulfilling the diagnosis of PAH. Whether these patients should be incorporated in group E is discussed below. The remaining patients had insufficient data in the CRF to be classifiable, pointing towards a limitation of the registry.

Of note, 6 patients were classified as group 5, having either segmental or complex CHD. This group has been abandoned in the most recent set of guidelines. This is a heterogenous group, with very different pathophysiologies leading to PH, including palliated single ventricle patients with elevated PVR (not included in the TOPP registry), segmental pulmonary hypertension and scimitar syndrome. Assembling such heterogenous conditions in a group with the view of inclusion in clinical trials and management recommendation would be nonsensical. However, patients with PH or pulmonary vascular disease and complex CHD are a growing population and cannot be ignored.

### Atrial septal defects

The causative role of ASD in PAH in children is debated. Natural history and haemodynamic studies show that pulmonary vascular disease rarely develops before the third or fourth decade of life (9, 23). Rare cases of PH in children with an ASD have been reported, although causality cannot be inferred in those often very young patients with several comorbidities and limited by incomplete haemodynamic data (24–26).

The lifetime risk of developing significant PH with an ASD has historically been assessed to be about 6%–10% (9, 27), but might be overestimated. Summarizing multiple haemodynamic studies on adult patients with ASD, Kulik et al. demonstrated that the vast majority of patients with significant (two to four times) increased pulmonary blood flow had normal or only modestly elevated PAP, even well into adulthood (11). Moreover, patients with PH (defined by a systolic PAP > 50 mmHg in the absence of invasive PVR and mPAP measurements in many studies) showed a marked decline in systolic PAP after ASD repair (11). In other words, the authors consider an ASD not sufficient to explain PAH in childhood and that is why we propose to classify these children as PAH-CHD group C. One hypothesis why PAH may nevertheless occur in children with ASD is that an additional trigger (e.g., endothelial inflammation) or genetic susceptibility may be required. In patients with PH and BPD, the influence of an atrial septal defect contributing to PH may be due to lung and pulmonary vascular hypoplasia. The identification of *SOX17* and *TBX4* mutations in children with PAH associated with ASD formerly thought to have early-onset Eisenmenger physiology corroborates this assumption (28, 29). Of note, these mutations have also been reported in some patients with a post-tricuspid shunt. Developmental lung disorders, associated with these mutations, could also play a role in early PH development.

Indeed, the poor survival in the current study of group C, composed by two third of ASD, appears different from survival of group B although this difference did not reach statistical significance across the full observational period. Survival curve of group C is very similar to iPAH, pointing toward a possible alternative etiology to PAH in patients with ASD. Moreover, survival of group C without ASD and group C with ASD was not different (Figure 3).

### Pulmonary hypertension after neonatal arterial switch operation

Pulmonary hypertension after repaired TGA was first described following atrial baffle surgery (30–32), and reported in 8%–20% of patients (33, 34). Atrial correction for transposition of the great arteries was often performed after 6 months of age, and many patients had a VSD. However, PH has also increasingly been reported following timely arterial switch operation performed in the neonatal period (21, 35, 36). Pulmonary arterial hypertension onset can be early or late after arterial switch operation, with an incidence roughly estimated at 0.6%–1% based on modest size selected cohort studies (12). Occurrence of PAH could well be underdiagnosed since most centres do not have a systematic screening program for PAH after arterial switch operation, or even misdiagnosed and considered in relation with proximal pulmonary branches stenosis.

Development of pulmonary vascular disease in TGA, especially in the absence of a significant shunt (VSD or PDA), remains of unclear mechanism but could involve prenatal hemodynamic alterations and abnormal distribution of oxygenated blood through the pulmonary vascular bed, leading to early development of pulmonary vascular disease (21). In utero constriction of the foramen ovale or PDA may also contribute. Because of the seemingly different cause of PAH in patients with repaired TGA, but also different patient's characteristics and disease course reported in the literature (younger age at onset, male preponderance, rapid deterioration), we decided to create a specific group E. There were only 7 patients from the registry in group E, and no death or transplant occurred during the follow-up period, limiting our capacity to draw conclusion on their clinical course.

However, increasing awareness of PAH following timely repair of TGA might allow this entity to be diagnosed more often, earlier, and better managed in the future.

Should we limit this new group E to repaired TGA, or should we include patients with corrected left-sided lesions and PAH? Indeed, some of the pathophysiological hypothesis described above may apply to patients with left-sided obstruction in their consequences on in-utero pulmonary vessel growth and development. We do not have sufficient data to answer this question, but this group of patients who never had a shunt is definitely a group that deserves to be addressed in the next set of guidelines.

### Ability of the classification to predict outcome in pediatric patients

Manes et al. reported different survival across clinical PAH-CHD subgroups from a large single centre, but the study population only included 16 pediatric patients (13). In a multicentre pediatric cohort including 134 PAH-CHD patients, (117 classified), no survival difference could be shown between the groups (8).

In the present study, analysing the outcome of the largest classifiable cohort of children with PAH-CHD so far, no statistically significant differences could be demonstrated in terms of survival between the groups. This could possibly be explained by the small number of patients in some subgroups and a relatively low frequency of death. The TOPP-2 registry, capturing data on follow-up of TOPP-1 participants who agreed to continue, as well as newly diagnosed PH pediatric patients, will increase patient's number and follow up, and may give some further insight into survival differences.

Nevertheless, a trend in the survival curves can be observed. Patients with coincidental shunts seem to have the worst prognosis, whereas Eisenmenger patients, despite higher mPAP and PVR hemodynamics, seem to have a better outcome. This finding is in line with previous pediatric and adult studies showing better outcome in Eisenmenger patients, explained by preserved right ventricular function, through partial relief of the right ventricular overload by right-to-left shunting and maintained fetal RV hypertrophy (37–39).

Our numbers are currently too low to observe a significant survival effect of relocating all ASDs in the coincidental group.

Survival of patients with PAH following timely TGA repair seems dismal in the literature (12). We were not able to confirm this due to the small number of patients in our group E cohort. Nonetheless, we believe that this is a distinct group of patients due to unique physiology. In the vast majority of patients any residual shunt due to an ASD, VSD, or PDA is closed within the first few days of life, so pulmonary overcirculation cannot be the cause of the PH.

### Limitations

This study has limitations inherent to those of an observational, non-interventional registry, with inhomogeneous quality of data. To overcome that, individual datasets were reviewed by two to three board members, in order to correctly classify the patients. Despite careful evaluation, data from 14 patients were insufficient to classify. Although TOPP is one of the largest international registries on pediatric PH, small number of patients and low frequency of events in some groups limited the possibility of comparing survival between groups. The requirement of a cardiac catheterization for registry inclusion may have provoked a selection bias, excluding milder cases. Outcome may have been altered by survival bias, with inclusion of prevalent and incident patients, although less problematic in pediatric population. Finally, our analysis did not capture patients potentially changing across ABCDE categories over time.

## Conclusion

Seventeen percent of children with PAH-CHD were unclassifiable using the conventional WSPH classification. The proposed modified classification addresses children with ASD and PAH and those with CHD without shunts. This modified classification was applicable to the registry cohort but failed to show a survival difference between categories. The classification of patients with CHD and PAH who never had a shunt, such as adequately corrected left-sided lesions, deserves further research.

## Data Availability

The original contributions presented in the study are included in the article/Supplementary Material, further inquiries can be directed to the corresponding authors.

## Author's note

Actelion/Janssen Pharmaceuticals Ltd does not participate in the management of the TOPP registry; neither does it have access to the database and the individual site and patient data. All decisions related to the Registry lie solely with the Executive Board of the Association for Pediatric Pulmonary Hypertension.
